# Aeroelastic flaps for dynamic airway stabilization: a novel approach to respiratory support

**DOI:** 10.1097/MS9.0000000000003521

**Published:** 2025-07-30

**Authors:** Muhammad Talha, Maliha Khalid, Muhammad Khan Buhadur Ali, Aminath Waafira

**Affiliations:** a King Edward Medical University, Lahore, Pakistan; b Jinnah Sindh Medical University, Karachi, Pakistan; c School of Medicine, The Maldives National University, Malé, Maldives

*To the Editor*,

In compliance with the TITAN guideline, this letter to the editor discusses the utilization, benefits, and prospects of utilization of aeroelastic flaps for respiratory support^[^1^]^. The dynamic stability of the human airway is central to effective respiration. Pathologies that cause airway collapse, including obstructive sleep apnea (OSA), chronic obstructive pulmonary disease (COPD), and neuromuscular respiratory dysfunction, affect millions of people. Among these, OSA alone affects nearly 1 billion persons in the world, with huge morbidity and mortality. Conventional means of airway stabilization, such as rigid mechanical supports and continuous positive airway pressure (CPAP), tend to be poorly tolerated because they fail to adjust to the natural variability of breathing patterns. The latest scientific advancement occurred in March 2025 with the inception of aeroelastic flaps—lightweight, adaptive structures that respond dynamically to airflow and pressure changes, thereby stabilizing the airway in an altogether different manner. This innovation departs radically from conventional methods and enters a dynamic solution that adopts the various phases of physiological breathing^[^2^]^.

Aeroelastic flaps borrow conceptual ideas from the aerospace field, where some flexible components of an aircraft wing adjust to aerodynamic forces for optimum stability and performance. These flaps were designed so that they passively deform when airflow through the respiratory tract occurs, dynamically providing support that moves with the patient’s respiratory cycle. Experimental studies have shown that such flexible flaps can eliminate flow disturbances and dampen oscillations by changing their shape in response to breathing, like the covert feathers lining bird wings, keeping aerodynamic robustness during low Reynolds numbers^[^3^]^. This biomimetic design could assist in counteracting airway collapse by keeping the passages open during both inspiration and expiration, therefore benefiting gas exchange and patient comfort.

Latest studies point towards the better performance of multi-segment aeroelastic flaps with continuous trailing edges and active shape control with better flow stability among various respiratory conditions^[^4^]^. These flaps implement sensor-based feedback systems, allowing for feedback on real-time stiffness and position adjustments to accommodate dynamic breathing patterns^[^5^]^. This adaptability, as opposed to rigid airway supports, translates to a possible reduced risk of tissue irritation and greater tolerance by patients. In addition, application of flutter suppression control technologies, adapted from aerospace systems, would optimize even further flap responsiveness and airway patency.

In clinical practice, the existence of airway collapse would lead to hypoxemia and hypercapnia along with an increased burden of morbidity and mortality due to respiratory diseases. CPAP therapy remains the conventional treatment for the disorder known as obstructive sleep apnea (OSA); however, the effectiveness of this intervention has been limited due to poor patient adherence because of discomfort and noise levels. Dynamic airway support that complements the natural act of respiration may be best offered in a targeted manner through aeroelastic flaps. An airway support solution dynamic to the airway disorders may align itself with regular breathing and thus ensure greater adherence and better health outcomes by reducing discomfort and improving mobility. Such flaps could easily be incorporated into existing strategies, either as pieces of wearable respiratory devices or as addenda to an endotracheal tube, greatly improving therapeutic efficacy while posing no challenge to mobility or comfort. However, well-defined clinical trials are essential for evaluating safety, efficacy, and long-term benefits in different patient populations who may also have co-morbidities such as COPD or neuromuscular disorders^[^6^]^.

In summary, the aeroelastic flaps represent a novel and very exciting technology for dynamic airway stabilization through biomimetics and aeroelasticity to provide adaptive respiratory support. The letter highlights an urgent need for deeper research into prototype fine-tuning, sophisticated feedback system integration, and large-scale clinical validation in establishing aeroelastic flaps as a game-changing technology in respiratory care. The axiom of patient welfare, above all, means that the technology might change the course of management of respiratory problems by improving outcomes while avoiding invasive or cumbersome mechanical supports.

## Data Availability

No data were generated for this manuscript.
